# Characterizing a Middle Bronze Palatial Wine Cellar from Tel Kabri, Israel

**DOI:** 10.1371/journal.pone.0106406

**Published:** 2014-08-27

**Authors:** Andrew J. Koh, Assaf Yasur-Landau, Eric H. Cline

**Affiliations:** 1 Departments of Classical Studies and Chemistry, Brandeis University, Waltham, Massachusetts, United States of America; 2 Department of Maritime Civilizations, The Leon H. Charney School of Marine Sciences and the Leon Recanati Institute of Maritime Studies, University of Haifa, Mount Carmel, Haifa, Israel; 3 Department of Classical and Near Eastern Languages and Civilizations, George Washington University, Washington, District of Columbia, United States of America; Chinese Academy of Sciences, China

## Abstract

Scholars have for generations recognized the importance of wine production, distribution, and consumption in relation to second millennium BC palatial complexes in the Mediterranean and Near East. However, direct archaeological evidence has rarely been offered, despite the prominence of ancient viticulture in administrative clay tablets, visual media, and various forms of documentation. Tartaric and syringic acids, along with evidence for resination, have been identified in ancient ceramics, but until now the archaeological contexts behind these sporadic discoveries had been uneven and vague, precluding definitive conclusions about the nature of ancient viticulture. The situation has now changed. During the 2013 excavation season of the Kabri Archaeological Project, a rare opportunity materialized when forty large storage vessels were found in situ in an enclosed room located to the west of the central courtyard within the Middle Bronze Age Canaanite palace. A comprehensive program of organic residue analysis has now revealed that all of the relatively uniform jars contain evidence for wine. Furthermore, the enclosed context inherent to a singular intact wine cellar presented an unprecedented opportunity for a scientifically intensive study, allowing for the detection of subtle differences in the ingredients or additives within similar wine jars of apparently the same vintage. Additives seem to have included honey, storax resin, terebinth resin, cedar oil, cyperus, juniper, and perhaps even mint, myrtle, or cinnamon, all or most of which are attested in the 18^th^ century BC Mari texts from Mesopotamia and the 15^th^ century BC Ebers Papyrus from Egypt. These additives suggest a sophisticated understanding of the botanical landscape and the pharmacopeic skills necessary to produce a complex beverage that balanced preservation, palatability, and psychoactivity. This new study has resulted in insights unachievable in the past, which contribute to a greater understanding not only of ancient viticulture but also of Canaanite palatial economy.

## Introduction

Tel Kabri is a 34-hectare site located in the western Galilee of modern-day Israel, five kilometers east of Nahariya. During the Middle Bronze Age (ca. 1900–1600 BC), the site was the center of a major Canaanite polity, with a palace covering at least 6,000 sq.m., making it the largest Middle Bronze Age palace excavated so far in Israel [1]–[3].

**Figure 1:**
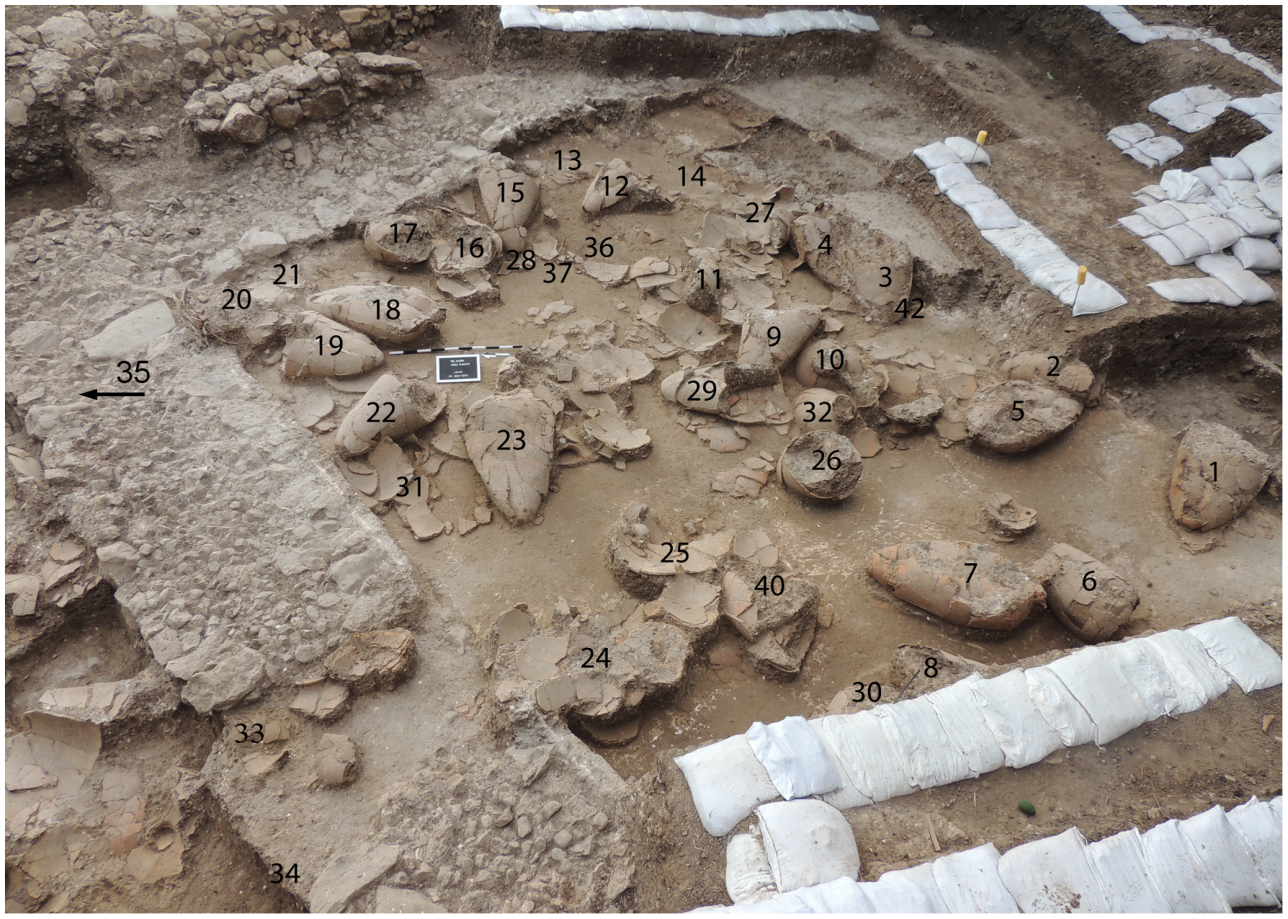
Kabri wine cellar with numbered jars (looking southeast).

**Figure 2:**
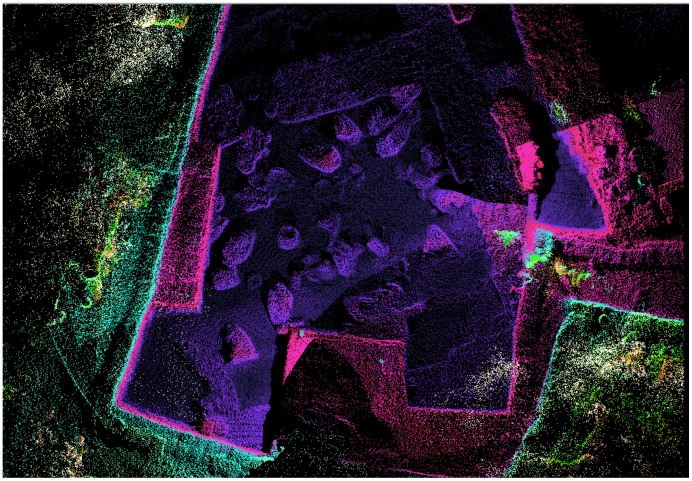
LiDAR map composed of millions of discrete points color coded to elevation.

During the 2013 excavations at Kabri, the remains of a palatial storage complex were uncovered, of which one room was excavated in its entirety. On the floor were the remains of approximately 40 restorable large, mostly handle-less, storage jars, as well as a few smaller vessels, all of which were found covered by a thick collapse of mudbricks from either the walls or the ceiling of the room (Figure 1). All of the fairly uniform, ca. 50-liter, storage jars were made from the most common type of ceramic fabric found at Kabri, from which the vast majority of other pottery in the palace was also made. It is probably related to Senonian marl, available locally as close as the nearby wadi. Inclusions include calcareous sand typical of the western Galilee. Before the systematic removal of the jars, samples from each were taken for organic residue analysis (ORA) and petrography; in addition, the fully articulated contents of the room were recorded using LiDAR, which collected millions of discrete three-dimensional data points and resulted in a surface plan accurate to within two mm (Figure 2).

Archaeologists have long identified wine as an important component of Bronze Age palatial economies, including social feasting [4]–[7]. Palatial wine storage rooms are known from documentary sources in both the Bronze Age Mediterranean and Mesopotamia, especially from the archives of the site of Mari [8], which are contemporary to the palace at Kabri. Their existence has also been further postulated on the basis of actual ceramic finds, including large storage jars in magazines, from Aegean palaces such as Pylos and Knossos, but none to date have been empirically confirmed by ORA. With numerous studies now verifying the efficacy and cost-effectiveness of analyzing ancient organic residues using gas chromatography in tandem with mass spectrometry (GC-MS) [9]–[12], Kabri's largely undisturbed palatial storage complex presented the ideal situation in which to conduct a comprehensive ORA program.

## Materials and Methods

All necessary permits were obtained for the described study, which complied with all relevant regulations. The Israel Antiquities Authority issued Permit G-42 to conduct excavations at Tel Kabri. No special permits were required for the present study. After the complete articulation of all jars, sherds of approximately the same size – small enough to sit flat in a 400 ml Griffin beaker – were identified near the base of each jar and immediately isolated in aluminum foil for subsequent residue extraction. The one exception was Jar 11, which had a second sample sherd taken from higher up on the body. Care was taken in documenting the entire procedure, so that precise LiDAR coordinates could be assigned to each sample sherd (Table 1). These sherds were then transported to the nearby Western Galilee Field School for non-destructive extraction using analytical grade ethanol in a process developed over the last ten years [9].

**Table 1:** Tartaric and syringic acid data from wine cellar jars with LiDAR coordinates of tested sherds.

ARCHEM #	Jar #	X,Y,Z LiDAR Coordinates (m)*	Tartaric Acid Absolute Abundance†	Tartaric Acid Relative Abundance (%)‡	Syringic Acid Absolute Abundance†	Syringic Acid Relative Abundance (%)‡
4324	1	65.391, 27.563, 51.131	3343756	57.79	202525	3.5
4296	2	67.219, 27.688, 50.978	3004788	37.50	239754	2.99
4295	3	67.953, 28.188, 50.961	13622358	62.24	196340	0.9
4293	4	68.469, 28.375, 51.018	7457540	55.61	135975	1.01
4323	5	66.297, 27.750, 51.082	2029624	35.25	75666	1.31
4312	6	65.578, 28.563, 51.041	145271	0.96	N/A	N/A
4304	7	65.656, 29.813, 50.930	404307	32.63	227830	18.39
4307	8	64.953, 29.500, 50.945	805717	18.39	N/A	N/A
4321	9	67.766, 29.313, 51.004	10324787	100	199528	1.93
4291	11	68.719, 29.250, 50.983	11985301	43.35	143509	0.52
4322	11	69.078, 29.313, 50.976	23144172	85.03	795193	2.92
4313	12	70.000, 29.875, 51.087	228412	100	3086	1.35
4316	13	70.688, 29.500, 51.091	1792475	9.02	N/A	N/A
4305	15	70.156, 30.750, 50.980	2294968	4.24	15457123	28.56
4300	16	69.578, 31.063, 50.980	391777	11.63	775446	23.02
4299	17	70.297, 31.813, 51.143	1303896	27.86	403613	8.63
4311	18	69.453, 32.375, 51.087	1708411	29.51	327871	5.66
4303	19	68.859, 32.125, 51.100	564888	3.77	7281238	48.66
4302	20	69.641, 33.188, 51.242	473817	5.28	298093	3.32
4297	22	67.828, 32.313, 51.111	546275	3.78	4539456	31.38
4306	23	67.219, 31.375, 50.981	774848	25.74	251112	8.34
4308	24	65.891, 31.875, 51.202	875524	32.06	382734	14.02
4310	25	66.688, 30.563, 50.870	756151	33.36	461805	20.37
4298	26	67.047, 29.563, 50.848	1188437	7.24	385049	2.35
4292	27	68.984, 28.563, 51.021	7053156	24.04	509446	1.74
4314	28	69.891, 30.438, 50.960	1862927	21.13	1399240	15.26
4320	29	67.625, 30.025, 50.870	4008053	18.79	711355	3.33
4301	31	67.500, 32.438, 50.993	1310401	1.67	635407	0.81
4317	33	66.672, 34.375, 50.949	5026543	100	445482	8.86
4325	34	65.719, 34.250, 50.792	5402354	19.9	199423	0.73
4319	35	68.109, 35.313, 50.828	491234	67.1	538802	73.59
4318	36	69.703, 30.188, 50.919	7921530	24.49	7415574	22.93
4315	37	69.500, 30.313, 50.909	995859	16.33	2792653	44.32
4289	Base	64.992, 27.375, 51.057	3548407	11.47	137261	0.44

*213400 and 768200 meters were removed from X and Y coordinates respectively for brevity.

† Peak area determined by integration in chromatograms.

‡ Percentage relative to maximum peak in a given chromatogram.

In addition to the insight gained into the efficacy of this non-destructive technique from both the current project as well as past studies [9], [13], [14], additional research is planned at Kabri to compare this technique with destructive techniques commonly used today [15]. Thus, adjacent sherds from the same vessels were collected in 2011 at Kabri with the explicit intent of comparing results from non-destructive and destructive extractions. Petrographic thin-section analysis is also being conducted by David Ben Shlomo of the Hebrew University. Our primary goal, however, is to illuminate patterns in palatial economies and social practices, some of which can only be studied through the contribution of ORA.

In all, 35 ORA samples from the storage room were ultimately extracted into filtered solution and stored in 20 ml scintillation vials; these samples came from 32 of the storage jars (with Jar 11 providing two samples), the base of a deep bowl or cup, and one soil sample to serve as a control (but which ultimately produced no organic residues). Sherds from the additional eight jars (of the 40) were collected but left untouched and unextracted as a control for future studies. The plan is to extract residues from two untested jars biannually to study the effects of sherd excavation with delayed extraction.

Less than two weeks after extraction, the 35 ORA samples were taken to the Brandeis University Department of Chemistry, concentrated to solid by rotary evaporator and redissolved in uninhibited THF to produce ∼2 ml GC-MS analytes, and injected into the university's newest GC-MS instrument – an Agilent 7890A GC with a HP-5MS column and a 5975C VL MSD Triple Axis Detector. The pulsed split injector and interface were both set to 250°C. The initial oven temperature was set to 100°C and held for two minutes before reaching 250°C at a rate of 10°C/min, at which time it was held for an additional 11 minutes, giving a total program time of 28 minutes/sample. Solvent blanks were intermittently utilized to verify that no contaminants existed from previous runs. Standard references of tartaric and syringic acids were ultimately produced (see below). The streamlined nature of the overall methodology allows for one person to take numerous samples from ground to instrument to interpretation in less than a month, which is considerably faster and more efficient than the norm.

## Results

**Figure 3:**
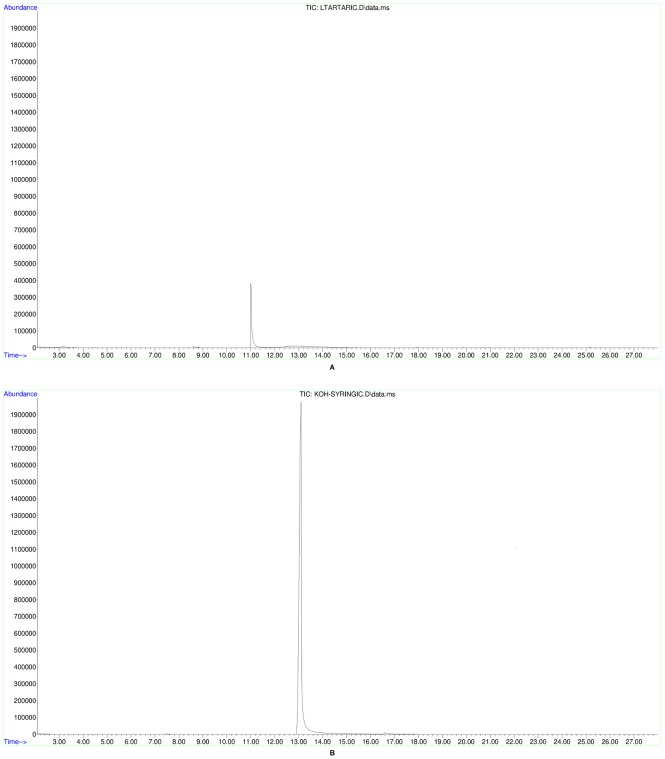
5 mM standard reference samples. A. Total-ion chromatogram of tartaric acid standard, B. Total-ion chromatogram of syringic acid standard.

**Figure 4:**
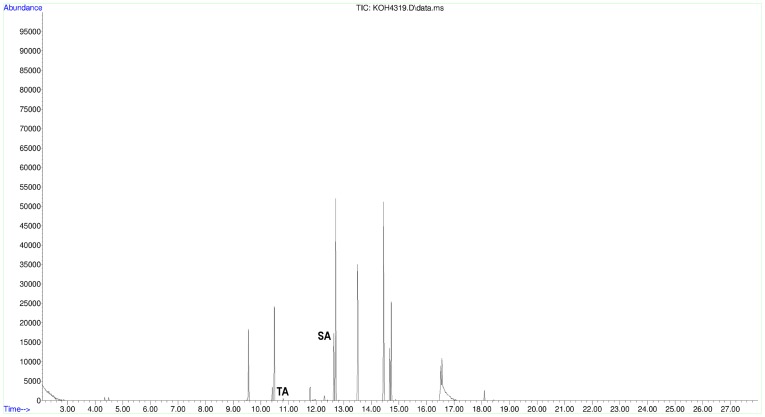
Representative total-ion chromatogram from Jar 35 (ARCHEM 4319).

**Figure 5:**
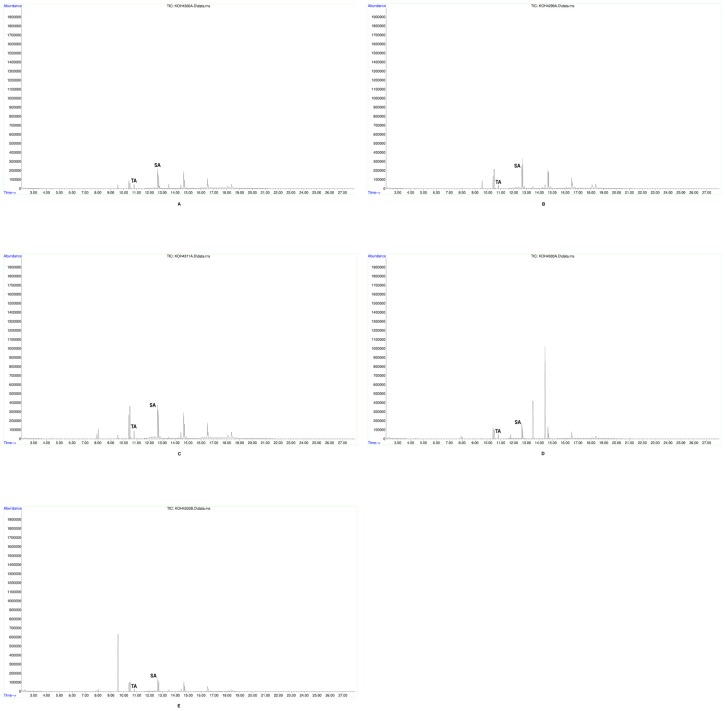
Total-ion chromatograms of Jars 16–20. A. Total-ion chromatogram from Jar 16 (ARCHEM 4300), B. Total-ion chromatogram from Jar 17 (ARCHEM 4299), C. Total-ion chromatogram from Jar 18 (ARCHEM 4311), D. Total-ion chromatogram from Jar 19 (ARCHEM 4303), E. Total-ion chromatogram from Jar 20 (ARCHEM 4302).

**Figure 6:**
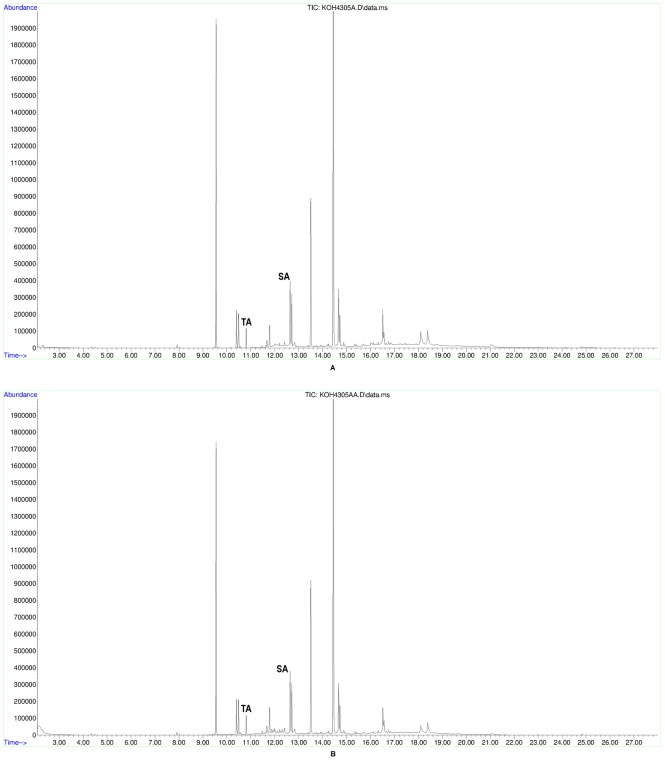
Verification of Jar 15 sample. A. Total-ion chromatogram from Jar 15 (ARCHEM 4305), B. Total-ion chromatogram from Jar 15 (ARCHEM 4305) verified.

All 32 tested jars and the small bowl/cup base contained tartaric acid after initial peak assignation using the National Institute of Standards and Technology mass spectral library, NIST 11 (Table 1). In order to verify this identification beyond a shadow of doubt, 5 mM standard reference samples (Figure 3) were prepared from commercially available standard compounds of both tartaric and syringic acids (Sigma-Aldrich), whose resultant spectra derived from the same GC-MS conditions were then manually cross-referenced with the ancient samples (Figure 4, 5, 6). All but three of the tested vessels also contained syringic acid and all but three contained cinnamic acid (Table 2). Cineole also was found in almost all of the tested vessels; it is absent from only four jars and the small bowl/cup base, which can readily be attributed to a lack of preservation. In addition, significant amounts of oleanoic acid were found in 27 of the tested jars, as well as the small bowl/cup base. Methyl syringate was found in 21 of the tested jars, while 19 contained cedrol.

**Table 2:** Chemical occurrence of additive compounds.

ARCHEM #	Jar #	Cinnamic acid	Cineole	Methyl Syringate	Cedrol	Oleanoic Acid	Moronic Acid	Masticadienoic Acid	Caryophyllene	Myrtenyl acetate
4324	1	◊								
4296	2	◊	◊	◊	◊	◊				
4295	3	◊	◊	◊		◊	◊	◊		
4293	4						◊	◊		
4323	5									
4312	6	◊	◊	◊	◊	◊				
4304	7		◊		◊	◊				
4307	8	◊	◊	◊		◊				
4321	9	◊	◊		◊	◊				
4291	11	◊					◊	◊		
4322	11	◊	◊	◊			◊	◊		
4313	12	◊	◊							
4316	13	◊	◊	◊	◊	◊				
4305	15	◊	◊	◊	◊	◊			◊	
4300	16	◊	◊	◊	◊	◊				◊
4299	17	◊	◊	◊	◊	◊				
4311	18	◊	◊	◊	◊	◊				
4303	19	◊	◊		◊	◊			◊	
4302	20	◊	◊	◊	◊	◊				
4297	22	◊	◊	◊	◊	◊				
4306	23	◊	◊	◊	◊	◊				
4308	24	◊	◊	◊		◊				
4310	25	◊	◊		◊	◊				
4298	26	◊	◊	◊	◊	◊				
4292	27	◊	◊	◊		◊	◊	◊		
4314	28	◊	◊	◊	◊	◊				
4320	29	◊	◊	◊		◊	◊	◊	◊	
4301	31	◊	◊		◊	◊				
4317	33	◊	◊		◊	◊			◊	
4325	34	◊		◊		◊	◊	◊		
4319	35	◊	◊			◊			◊	
4318	36	◊	◊	◊		◊	◊	◊	◊	
4315	37	◊	◊		◊	◊				
4289	Base	◊		◊		◊	◊	◊	◊	

**Figure 7:**
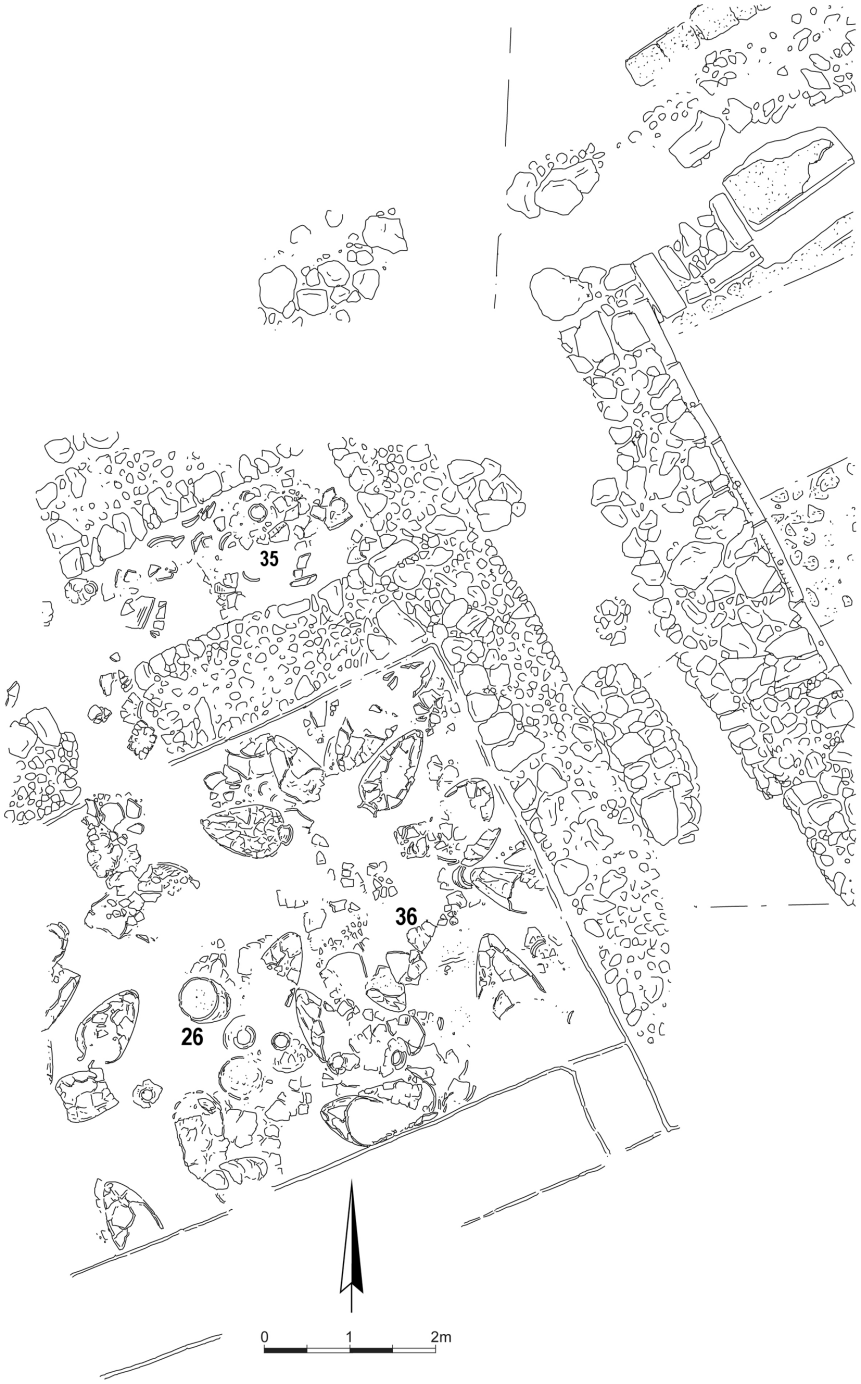
Plan of Kabri wine cellar.

The fact that the ca. 3 m.×1 m. eastern antechamber floor (Figure 7) is the lowest part of the study area but produced excellent ORA results mitigates fears of preservation being obscured by any pooling groundwater. In addition, note again that Jar 11 was tested in two locations on the jar – one near the base like all the other samples and one quite a bit higher up on the body. While this second, less optimal, location higher up on the body did not prevent the detection of both tartaric and syringic acids (Table 1), the second sample's peak abundances were noticeably lower and at least two potentially diagnostic compounds were not detected at all (Table 2). This has obvious ramifications concerning the location of a sampled sherd on a vessel, a variable that is often ignored and even left unnoted, especially when only a few vessel sherds are extant.

### Interpretation

Every sample taken from the vessels that were tested yielded positive results (Table 2). The nature and preservation of the room in conjunction with the swift and careful isolation of sampled sherds while in the field, followed by immediate extraction, likely contributed to the high rate of success and excellent data produced, which stand in contrast to uneven results in the past from objects typically excavated decades into the past and housed in uncertain conditions.

As noted, all 32 tested jars and the small bowl/cup base contained tartaric acid, and all but three also contained syringic acid. Combined with our knowledge of vessel typology and palatial economies, the presence of both tartaric and syringic acids in relative abundance as biomarkers indicates that all of these vessels originally held wine [9]–[12], [16] and that we may be confident in identifying this space as a wine storage room – that is to say, a wine cellar. On a related note, we should mention that, in addition to vineyards reestablished during the 19^th^ century in the viticulture-friendly Upper Galilee by Baron Edmond de Rothschild using grape varieties imported from Bordeaux, we know from a papyrus in the Zenon Archive from Ptolemaic Egypt dating to 257 BC (p.lond.7.1948) that the ancient Bethanath estate located just 15 km to the southeast (modern Bi'ina outside Karmiel in the Beit HaKerem Valley) produced wine from 80,000 vines, which was purportedly indistinguishable from the celebrated wines of Chios [17]. Salvage excavations in 2001 at the adjacent tell of Nahf led excavators to conclude that the area was an important center for viticulture certainly in the Hellenistic period and perhaps as far back as the Early Bronze Age IB period (ca. 3100 BC), judging by the finds [18]. Supported by the locally-sourced clay of the Kabri wine jars, this makes Karmiel a good candidate for the location of Kabri's ancient vineyards. If grape DNA can be isolated at Kabri in future seasons, it is possible that more closely related cultivars that are presently feral in the region [19] or surviving in European vineyards after export in antiquity [20] could be identified or even cloned.

The lack of syringic acid in three of the tested jars opens up the possibility that those three held white wine, rather than red wine, but it is difficult to say with certainty without further evidence, such as distinguishing markings on the jars. It is possible that the syringic acid did not survive or was undetectable in these three jars during the present study.

In addition, all but three of the tested jars contained cinnamic acid. While it can be notoriously difficult to match extant compounds to their original sources from antiquity, and there are alternatives for each suggested here, it is possible to present likely sources based on 1) concentrations of compounds inherently found in commodities; 2) ethnobotanical knowledge of the natural distribution of ancient commodities; and 3) surviving documentary accounts of commodities acquisition and utilization. As new internal and external evidence is produced, these interpretations can be duly updated. In the case of cinnamic acid, it occurs foremost in the storax resin of *Liquidambar orientalis*, or Oriental Storax or Sweetgum, at 150,000 ppm if one discounts its New World kin, *L. styracifula*, at 230,000 ppm. Cinnamic acid also occurs in *Styrax officinalis*, or Styrax or Snowbell, whose benzoe resin is chemically similar and presumably explains historical descriptions of “storax” that more closely resemble it than the resin from *Liquidambar*. One possible clue as to its source at Kabri is the occurrence of oleanoic acid in *Liquidambar* and *Pistacia*, but not *Styrax* [21]. Due to its prevalence in the Kabri wine jars, it is probable that this aromatic resin was the primary preservative added at nearby wine production centers in the Upper Galilee region, still renowned for viticulture to this day, before transport to the palace.

Cineole also was found in all but four of the tested jars. Isolating the source(s) of cineole can be difficult, but likely candidates include cyperus roots (*Cyperus rotundus*), mint (*Mentha*), juniper berries (*Juniper communis* or *phoenicea*) well known today for the connection to gin, and cinnamon bark (*Cinnamomum*) [22]. It is notable that all of these potential candidates were also ingredients of Egyptian kyphi, whose herbal additives have been postulated to originate from the Levant [10]. In addition, caryophyllene was found in six of these jars as well, which supports the presence of cedar oil (*Cedrus libani*), mint, juniper berries, and cinnamon bark as additives.

Methyl syringate, which was found in 21 of the tested jars, occurs in good quantity in honey [23]. It would not be at all surprising if honey had been added to this wine, for it fits well with the textual evidence from both the 18^th^ century BC Mari tablets [8] and Egyptian kyphi recipes [24] from the 15^th^ century BC onwards. Presuming that this physical evidence at Kabri is indicative of local apiculture, it would push back direct evidence in the southern Levant by at least seven centuries before the apiary at Tel Rehov [25]. Nineteen of the tested jars contained cedrol, which likely originated from the nearby stands of *C. libani* (Cedar of Lebanon) and its cedar oil [26] or, less likely, from juniper, where it only occurs in small quantities (2000 ppm).

The oleanoic acid that was found in 27 of the tested jars, as well as the small bowl/cup base, could have come from terebinth (*Pistacia palaestina or terebinthus*) resin, a local antimicrobial additive [26] long associated with ancient wines [11]. This interpretation is supported by the detection of moronic and masticadienoic acids in five of the tested jars, in addition to the small bowl/cup base. However, despite robust quantities of oleanoic acid in the other 22 tested jars, the lack of these two additional triterpenes – moronic and masticadienoic acid – in these jars leaves open the possibility that their oleanoic acid derives from a different source, such as the aforementioned storax resin or cyperus.

Overall, the five tested jars with the best represented organic residues – judging by the definition and number of individual GC peaks – occurred in two general areas of the storage room: in the eastern antechamber near the northern entrance, exemplified by intact Jar 35 (Figure 4) and towards the south central part of the room between Jars 26 and 36 (Figure 7, Table 2). This latter group was found near a feature/platform abutting the southern wall and perhaps not coincidentally surrounding an installation ensconced in the ground in front of the feature/platform and found under Jar 26. These conditions combined with jars producing simpler and noticeably consistent chromatograms on the cellar's east periphery (Figure 5, 6) suggest that wines may have been brought into the cellar from the southeast, triaged near the east wall, treated at the central installation, and stored in the northern antechambers before consumption, although this awaits final verification after the entire building is published. Jars 15–20 with their simpler and consistent chromatograms represent a line of well-preserved vessels that might have originally leaned against one another and the east wall of the wine cellar awaiting treatment at the central installation. Like Jar 11, Jar 15 received slightly special attention from us. In the latter's case, two subsamples from the jar's master sample were injected as bookends into the Brandeis GC-MS approximately twelve hours apart with samples from other jars intervening with the usual blanks interspersed throughout. In the end, these two subsamples produced nearly indistinguishable chromatograms (Figure 6).

## Discussion

Tartaric acid and syringic acid have been identified in earlier production and funerary deposits [10], [12], but the archaeological contexts behind these sporadic discoveries had been limited, precluding definitive conclusions about the nature of ancient viticulture, especially as it pertains to consumption. The storage jars that we found in the closed and sealed archaeological context within the well-studied palace at Kabri are unlikely to have held anything but liquids, considering their narrow necks, and the ORA conducted on 32 of the 40 (as mentioned above, sherds from the additional eight jars were collected but left untouched and unextracted as a control for future studies) has now allowed us to confidently identify them as belonging to the oldest and largest palatial wine cellar that has been chemically confirmed from the ancient Near East. Moreover, the controlled context containing numerous similar wine jars of presumably the same vintage presented an unprecedented opportunity for a scientifically intensive study of its organic residue remains, allowing for the detection of subtle differences in the quality and quantity of ingredients or additives within the various wine jars, though the possibility that the jars were used and reused over the course of their lifetime must also be considered.

Overall, the ORA indicates that the Kabri palatial wine cellar included resinated red wine, and possibly resinated white wine, with many of the jars containing herbal additives in fairly consistent ratios to both tartaric and syringic acids (Table 3). These ingredients, of which only trace compounds like oleanoic acid are now extant, may have included those mentioned above, such as honey, storax resin, terebinth resin, cyperus, cedar oil, juniper, and perhaps even mint, myrtle (*Myrtus communis*) [26], or cinnamon. Many of these ingredients, like the honey discussed above, are attested as additives to wine in the 18^th^ century BC Mari texts from Mesopotamia and in Egyptian kyphi recipes published for at least two millennia starting by the 15^th^ century BC.

**Table 3:** Additives data from wine cellar jars.

ARCHEM #	Jar #	Compounds of Interest	Possible Additive Source(s)	Absolute Abundance*	Relative Abundance (%)†	Ratio to Tartaric Acid‡	Ratio to Syringic Acid‡
4324	1	cinnamic acid	storax resin	1494759	25.83	0.4470	7.3806
4296	2	cinnamic acid	storax resin	1276725	15.93	0.4249	5.3251
		cineole	cyperus, mint, juniper berries, or cinnamon bark	228932	2.86	0.0762	0.9549
		methyl syringate	honey	158663	1.98	0.0528	0.6618
		oleanoic acid	cyperus, pistacia resin, storax resin	1614423	20.15	0.5373	6.7337
		cedrol	cedar oil	2043677	25.50	0.6801	8.5241
4295	3	cinnamic acid	storax resin	5288827	24.17	0.3882	26.9371
		cineole	cyperus, mint, juniper berries, or cinnamon bark	97827	0.45	0.0072	0.4983
		methyl syringate	honey	135629	0.62	0.0100	0.6908
		moronic Acid	pistacia resin	4781078	21.85	0.3510	24.3510
		oleanoic acid	cyperus, pistacia resin, storax resin	143789	0.66	0.0106	0.7323
		masticadienoic acid	pistacia resin	196814	0.90	0.0144	1.0024
4293	4	moronic acid	pistacia resin	2566950	19.14	0.3442	18.8781
		masticadienoic acid	pistacia resin	157914	1.18	0.0212	1.1613
4323	5	N/A	N/A	N/A	N/A	N/A	N/A
4312	6	cinnamic acid	storax resin	41939	0.28	0.2887	N/A
		cineole	cyperus, mint, juniper berries, or cinnamon bark	4251964	28.07	29.2692	N/A
		methyl syringate	honey	539556	3.56	3.7141	N/A
		oleanoic acid	cyperus, pistacia resin, storax resin	4528939	29.90	31.1758	N/A
		cedrol	cedar oil	366042	2.42	2.5197	N/A
4304	7	cineole	cyperus, mint, juniper berries, or cinnamon bark	26747	2.14	0.0662	0.1174
		oleanoic acid	cyperus, pistacia resin, storax resin	1239199	100	3.0650	5.4391
		cedrol	cedar oil	121888	9.84	0.3015	0.5350
4307	8	cinnamic acid	storax resin	4379377	100	5.4354	N/A
		cineole	cyperus, mint, juniper berries, or cinnamon bark	19364	0.44	0.0240	N/A
		methyl syringate	honey	208538	4.76	0.2588	N/A
		oleanoic acid	cyperus, pistacia resin, storax resin	390542	8.92	0.4847	N/A
4321	9	cinnamic acid	storax resin	2442191	23.65	0.2365	12.2398
		cineole	cyperus, mint, juniper berries, or cinnamon bark	573568	5.56	0.0556	2.8746
		oleanoic acid	cyperus, pistacia resin, storax resin	1018503	9.86	0.0986	5.1046
		cedrol	cedar oil	196248	1.90	0.0190	0.9836
4291	11	cinnamic acid	storax resin	245035	0.89	0.0204	1.7075
		moronic acid	pistacia resin	4975001	18.00	0.4151	34.6668
		masticadienoic acid	pistacia resin	470967	1.70	0.0393	3.2818
4322	11	cinnamic acid	storax resin	2021760	7.43	0.0874	2.5425
		cineole	cyperus, mint, juniper berries, or cinnamon bark	96850	0.36	0.0042	0.1218
		methyl syringate	honey	105724	0.39	0.0046	0.1330
		moronic acid	pistacia resin	9005507	33.08	0.3891	11.3249
		masticadienoic acid	pistacia resin	791283	2.91	0.0342	0.9951
4313	12	cinnamic acid	storax resin	7282	3.19	0.0319	2.3597
		cineole	cyperus, mint, juniper berries, or cinnamon bark	34235	14.99	0.1499	11.0936
4316	13	cinnamic acid	storax resin	19864640	100	11.0822	N/A
		cineole	cyperus, mint, juniper berries, or cinnamon bark	2418428	12.17	1.3492	N/A
		methyl syringate	honey	1206024	6.07	0.6728	N/A
		oleanoic acid	cyperus, pistacia resin, storax resin	5158279	25.97	2.8777	N/A
		cedrol	cedar oil	3763042	18.94	2.0994	N/A
4305	15	cinnamic acid	storax resin	28785576	53.19	12.5429	1.8623
		cineole	cyperus, mint, juniper berries, or cinnamon bark	3235294	5.98	1.4097	0.2093
		caryophyllene	cedar oil, mint, juniper berries, or cinnamon bark	2652198	4.90	1.1557	0.1716
		methyl syringate	honey	1289913	2.38	0.5621	0.0835
		oleanoic acid	cyperus, pistacia resin, storax resin	5544408	10.24	2.4159	0.3587
		cedrol	cedar oil	7012114	12.96	3.0554	0.4536
4300	16	cinnamic acid	storax resin	555136	16.48	1.4170	0.7159
		cineole	cyperus, mint, juniper berries, or cinnamon bark	1250074	37.11	3.1908	1.6121
		methyl syringate	honey	311317	9.24	0.7946	0.4015
		myrtenyl acetate	myrtle	90137	2.68	0.2301	0.1162
		oleanoic acid	cyperus, pistacia resin, storax resin	2957384	87.80	7.5486	3.8138
		cedrol	cedar oil	2898403	86.05	7.3981	3.7377
4299	17	cinnamic acid	storax resin	1162478	24.84	0.8915	2.8802
		cineole	cyperus, mint, juniper berries, or cinnamon bark	3030522	64.76	2.3242	7.5085
		methyl syringate	honey	283333	6.05	0.2173	0.7020
		oleanoic acid	cyperus, pistacia resin, storax resin	2812546	60.10	2.1570	6.9684
		cedrol	cedar oil	1838714	39.29	1.4102	4.5556
4311	18	cinnamic acid	storax resin	577577	9.98	0.3381	1.7616
		cineole	cyperus, mint, juniper berries, or cinnamon bark	5161095	89.14	3.0210	15.7412
		methyl syringate	honey	965568	16.68	0.5652	2.9450
		oleanoic acid	cyperus, pistacia resin, storax resin	4619771	79.79	2.7041	14.0902
		cedrol	cedar oil	1094098	18.90	0.6404	3.3370
4303	19	cinnamic acid	storax resin	53410	0.36	0.0945	0.0073
		cineole	cyperus, mint, juniper berries, or cinnamon bark	1712400	11.44	3.0314	0.2352
		caryophyllene	cedar oil, mint, juniper berries, or cinnamon bark	766913	5.13	1.3576	0.1053
		oleanoic acid	cyperus, pistacia resin, storax resin	1987962	13.29	3.5192	0.2730
		cedrol	cedar oil	735800	4.92	1.3026	0.1011
4302	20	cinnamic acid	storax resin	8975654	100	18.9433	30.1102
		cineole	cyperus, mint, juniper berries, or cinnamon bark	149823	16.49	0.3162	0.5026
		methyl syringate	honey	155327	1.73	0.3278	0.5211
		oleanoic acid	cyperus, pistacia resin, storax resin	1613479	17.98	3.4053	5.4127
		cedrol	cedar oil	440547	4.91	0.9298	1.4779
4297	22	cinnamic acid	storax resin	14466863	100	26.4827	3.1869
		cineole	cyperus, mint, juniper berries, or cinnamon bark	879020	6.08	1.6091	0.1936
		methyl syringate	honey	144339	1.00	0.2642	0.0318
		oleanoic acid	cyperus, pistacia resin, storax resin	1913842	13.23	3.5034	0.4216
		cedrol	cedar oil	898342	6.21	1.6445	0.1979
4306	23	cinnamic acid	storax resin	583336	19.38	0.7528	2.3230
		cineole	cyperus, mint, juniper berries, or cinnamon bark	1616217	53.68	2.0859	6.4362
		methyl syringate	honey	160411	5.33	0.2070	0.6388
		oleanoic acid	cyperus, pistacia resin, storax resin	2465266	81.88	3.1816	9.8174
		cedrol	cedar oil	1467052	48.73	1.8933	5.8422
4308	24	cinnamic acid	storax resin	88711	3.25	0.1013	0.2318
		cineole	cyperus, mint, juniper berries, or cinnamon bark	2416827	88.51	2.7604	6.3146
		methyl syringate	honey	212555	7.78	0.2428	0.5554
		oleanoic acid	cyperus, pistacia resin, storax resin	2090629	76.56	2.3879	5.4624
4310	25	cinnamic acid	storax resin	8059	0.36	.0107	0.0044
		cineole	cyperus, mint, juniper berries, or cinnamon bark	215071	9.49	0.2844	0.1183
		oleanoic acid	cyperus, pistacia resin, storax resin	2266978	100	2.9980	1.2471
		cedrol	cedar oil	1281799	56.54	1.6952	0.7051
4298	26	cinnamic acid	storax resin	16405292	100	13.8041	42.6057
		cineole	cyperus, mint, juniper berries, or cinnamon bark	1974529	12.04	1.6615	5.1280
		methyl syringate	honey	741031	4.52	0.6235	1.9245
		oleanoic acid	cyperus, pistacia resin, storax resin	3827611	23.33	3.2207	9.9406
		cedrol	cedar oil	254127	15.53	0.2138	0.6600
4292	27	cinnamic acid	storax resin	243079	0.83	0.0345	0.4771
		cineole	cyperus, mint, juniper berries, or cinnamon bark	64891	0.22	0.0092	0.1274
		methyl syringate	honey	104826	0.36	0.0149	0.2058
		moronic acid	pistacia resin	5290463	18.03	0.7501	10.3847
		oleanoic acid	cyperus, pistacia resin, storax resin	105802	0.36	0.0150	0.2077
		masticadienoic acid	pistacia resin	389893	1.33	0.0553	0.7653
4314	28	cinnamic acid	storax resin	1701520	19.30		
		cineole	cyperus, mint, juniper berries, or cinnamon bark	3946414	44.76	2.1184	2.8204
		methyl syringate	honey	1578962	17.91	0.8476	1.1284
		oleanoic acid	cyperus, pistacia resin, storax resin	7184739	81.48	3.8567	5.1347
		cedrol	cedar oil	4705967	53.37	2.5261	3.3632
4320	29	cinnamic acid	storax resin	894407	4.19	0.2232	1.2573
		cineole	cyperus, mint, juniper berries, or cinnamon bark	105494	0.49	0.0263	0.1483
		caryophyllene	cedar oil, mint, juniper berries, or cinnamon bark	234542	1.10	0.0585	0.3297
		methyl syringate	honey	200929	0.94	0.0501	0.2825
		moronic acid	pistacia resin	5538087	25.96	1.3817	7.7853
		oleanoic acid	cyperus, pistacia resin, storax resin	145885	0.68	0.0364	0.2051
		masticadienoic acid	pistacia resin	465817	2.18	0.1162	0.6548
4301	31	cinnamic acid	storax resin	78383225	100	59.8162	123.3591
		cineole	cyperus, mint, juniper berries, or cinnamon bark	3412391	4.35	2.6041	5.3704
		oleanoic acid	cyperus, pistacia resin, storax resin	5089707	6.49	3.8841	8.0102
		cedrol	cedar oil	3385811	4.32	2.5838	5.3286
4317	33	cinnamic acid	storax resin	2209658	43.96	0.4396	4.9602
		cineole	cyperus, mint, juniper berries, or cinnamon bark	280319	5.58	0.0558	0.6292
		caryophyllene	cedar oil, mint, juniper berries, or cinnamon bark	107570	2.14	0.0214	0.2415
		oleanoic acid	cyperus, pistacia resin, storax resin	560737	11.16	0.1116	1.2587
		cedrol	cedar oil	41459	0.82	0.0082	0.0931
4325	34	cinnamic acid	storax resin	537645	1.98	0.0995	2.6960
		methyl syringate	honey	35184	0.13	0.0065	0.1764
		moronic acid	pistacia resin	3838164	14.14	0.7105	19.2463
		oleanoic acid	cyperus, pistacia resin, storax resin	59010	0.22	0.0109	0.2959
		masticadienoic acid	pistacia resin	303105	1.12	0.0561	1.5199
4319	35	cinnamic acid	storax resin	246579	33.68	0.5020	0.4576
		cineole	cyperus, mint, juniper berries, or cinnamon bark	295643	40.38	0.6018	0.5487
		caryophyllene	cedar oil, mint, juniper berries, or cinnamon bark	46537	6.36	0.0947	0.0864
		oleanoic acid	cyperus, pistacia resin, storax resin	150258	20.52	0.3059	0.2789
4318	36	cinnamic acid	storax resin	2605220	8.05	0.3289	0.3513
		cineole	cyperus, mint, juniper berries, or cinnamon bark	3147852	9.73	0.3974	0.4245
		caryophyllene	cedar oil, mint, juniper berries, or cinnamon bark	888008	2.75	0.1121	0.1197
		methyl syringate	honey	240911	0.74	0.0304	0.0325
		moronic acid	pistacia resin	8034511	24.84	1.0143	1.0835
		oleanoic acid	cyperus, pistacia resin, storax resin	238757	0.74	0.0301	0.0322
		masticadienoic acid	pistacia resin	338245	1.05	0.0427	0.0456
4315	37	cinnamic acid	storax resin	1296918	21.27	1.3023	0.4644
		cineole	cyperus, mint, juniper berries, or cinnamon bark	391683	6.42	0.3933	0.1403
		oleanoic acid	cyperus, pistacia resin, storax resin	2582576	42.35	2.5933	0.9248
		cedrol	cedar oil	1654328	27.13	1.6612	0.5924
4289	Base	cinnamic acid	storax resin	6555436	21.19	1.8474	47.7589
		caryophyllene	cedar oil, mint, juniper berries, or cinnamon bark	1308486	4.23	0.3688	9.5328
		methyl syringate	honey	228195	0.74	0.0643	1.6625
		moronic acid	pistacia resin	10566264	34.15	2.9777	76.9794
		oleanoic acid	cyperus, pistacia resin, storax resin	207828	0.67	0.0586	1.5141
		masticadienoic acid	pistacia resin	333968	1.08	0.0941	2.4331

*Peak area determined by integration in chromatograms.

† Percentage relative to maximum peak in a given chromatogram.

‡ Ratio of compound of interest's absolute abundance to organic acid's absolute abundance.

The Mari texts generally record types of wine and additives; for instance, one mentions “One jar of strong wine, one jar of sweet wine, and eight jars of wine of second quality shipped together with three types of herbal aromatics: one kirippum-jar of oil of Cyprus, one kirippum-jar of oil of myrtle, and one kirrippum-jar of oil of juniper” [8]. The kyphi texts are a bit more involved. For instance, after recounting the methodical brewing process of kyphi documented by the Egyptian priest Manetho in the 3^rd^ century BC, the 1^st^ century Greek historian Plutarch remarks that kyphi was used as a potion to cleanse internal organs [27]. Besides the obvious antimicrobial properties of many of these additives [28], some like cedar oil were likely known centuries before the Middle Bronze Age to possess astringent, diuretic, sedative, and stimulant properties as well [29]. The complex recipe of the Kabri wine thus may provide concrete evidence for the sophistication of Canaanite viticulture.

Furthermore, the large total volume of the stored wine – up to 2,000 liters – and the context of this storeroom, next to a ceremonial room within the palace in which banquets might have been held, may contribute to a greater understanding of Canaanite court ceremony and economy. Although 2,000 liters – or the equivalent of 3,000 modern bottles of wine – may seem like a lot, it is not enough for wide-spread distribution and should probably be seen as directly related to consumption within the palace rather than to either production or distribution [30]; in other words, we may have here the private reserve of the ruler and his household. When considered with issues of long-term preservation in antiquity and the overall consistency of both the wine and containers, it seems likely that the wine cellar held a single vintage, which was habitually replenished in a given year.

### Future Work

In looking to the future, we note that there is a southeastern entrance (or exit) to this room. This connects the excavated storage room with another, as yet largely unexcavated, room that is located directly to the south, in which the remains of at least six additional large storage jars have already been found. These were excavated and removed at the end of the 2013 season, since they would not have survived the winter. There is also what appears to be yet another opening, this one leading off to the northwest of our storage room, which may lead to additional rooms in this storage complex, but investigation of these additional areas will have to wait until the next excavation season, in 2015.
